# The Diagnostic and Therapeutic Challenges of Posttraumatic Iris Implantation Cysts: Illustrative Case Presentations and a Review of the Literature

**DOI:** 10.1155/2015/375947

**Published:** 2015-08-12

**Authors:** Nandini Venkateswaran, Steven S. T. Ching, William Fischer, Frank Lee, Gabrielle Yeaney, Holly B. Hindman

**Affiliations:** ^1^University of Rochester School of Medicine and Dentistry, University of Rochester, Rochester, NY 14642, USA; ^2^Flaum Eye Institute, University of Rochester School of Medicine and Dentistry, Rochester, NY 14642, USA; ^3^Department of Pathology and Laboratory Medicine, University of Rochester School of Medicine and Dentistry, Rochester, NY 14642, USA; ^4^Center for Visual Science, University of Rochester, Rochester, NY 14627, USA

## Abstract

Posttraumatic iris implantation cysts are rare ocular findings that are often associated with poor visual outcomes. Iris implantation cysts can present clinicians with diagnostic and therapeutic challenges given their variable presentations and frequently destructive nature. In this paper, we provide descriptions of two unusual cases of posttraumatic iris implantation cysts. The first case is of a recurrent keratin-filled iris implantation cyst that developed after open globe injury and intraocular implantation of cilia and was treated with cyst debulking procedures, injections of 5-Fluorouracil, and iridocyclectomy. The second case is of recurrent posttraumatic serous iris implantation cysts that were treated with laser, cyst aspiration, and injections of 5-Fluorouracil. We use these cases as a platform to discuss the different manifestations of implantation cysts, the roles of anterior segment optical coherence tomography, ultrasound biomicroscopy, and histopathology in facilitating timely and accurate diagnosis and review the range of available therapeutic modalities. We discuss conservative treatment approaches, including the novel use of 5-Fluorouracil therapy as an adjunct therapy, as well as more aggressive surgical excision requiring ocular reconstruction. Through a discussion of these cases and review of the literature, we provide recommendations to assist clinicians in managing this uncommon but vision-threatening condition and minimizing complications.

## 1. Introduction

Posttraumatic or postsurgical iris implantation cysts, a relatively rare occurrence, present the clinician with several challenges. The clinician is required to accurately determine the pathogenesis of these cysts as well as decide upon the appropriate timing and modality of treatment. Iris cysts are classified as primary or secondary cysts based upon their etiology [1–3]. Primary cysts include posterior pigment epithelial cysts, iris stromal cysts, and free-floating/dislodged cysts while secondary or acquired cysts develop in the setting of ocular trauma, tumors, inflammatory conditions, parasitic ocular invasion, or prolonged use of topical miotics or prostaglandins [1, 2, 4]. Among secondary cysts, posttraumatic implantation cysts are encountered more commonly in clinical practice. They are particularly difficult to manage, given their variable presentations and risk for complications, and are also often associated with poor visual outcomes [1–5]. In this paper, we highlight two unusual cases of recurrent iris implantation cysts with varying etiologies, presentations, and treatment approaches and use them as a platform to review and discuss the diagnostic and therapeutic challenges faced when addressing this uncommon condition.

## 2. Case 1

**Figure 1:**
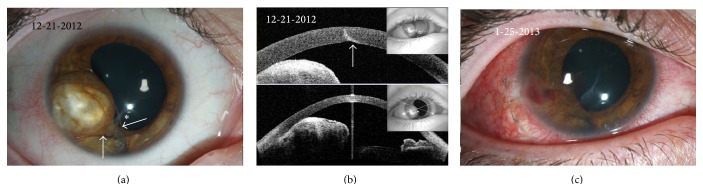
Clinical and diagnostic images from case 1: initial presentation. (a) Slit-lamp photograph of the patient's right eye upon presentation two years after his initial penetrating ocular injury with a wire fence. An inferocentral corneal scar is seen (asterisk). The pupil is irregular with a large, opaque, iris cyst extending from 7 to 9 o'clock with two embedded cilia (arrows). The lens is clear with no signs of cataract formation. (b) Anterior segment optical coherence tomography (AS-OCT) images illustrating the healed corneal laceration (arrow) from the patient's prior penetrating ocular injury and the location and contours of the iris implantation cyst. The cyst abuts the posterior cornea and there is absence of normal angle architecture. High reflectivity within the cyst indicates that the lesion is unlikely to be fluid-filled. (c) Slit-lamp photograph of the patient's eye one week after debulking of the cyst and injection of 5-Fluorouracil (5-FU) within the cyst cavity. The cyst was approached through a temporal scleral tunnel incision to facilitate precise entry into the body of the cyst for drainage and prevent expulsion of debris into the anterior chamber. Following surgery, the cyst initially resolved and there is rounding of the pupil.

A 22-year-old male with a history of penetrating ocular trauma with a wire fence in the right eye underwent full-thickness corneal laceration and iris prolapse repair at an outside institution in 2010. He first presented to the Flaum Eye Institute in 2012 with an enlarging “white spot” in the previously injured right eye, discoloration of the iris, and symptoms of chronic pain, photophobia, and epiphora. Visual acuity was 20/150 in the right eye, intraocular pressure was 14 mmHg, and anterior segment exam revealed a 4 mm healed central corneal scar, quiet anterior chamber, and a large iris cyst from 7 to 9 o'clock with 2 cilia embedded in the iris (Figures 1(a) and 1(b)). Over the next month, he experienced progressive growth of the cyst and worsening of his symptoms and subsequently underwent debulking of the iris cyst, explanation of both cilia, and injection of 1000 mcg 5-Fluorouracil (5-FU) in 0.2 cc of Viscoat into the cyst cavity. Gram stain and aerobic culture of the aspirated material revealed no organisms and pathological analysis revealed benign nonpigmented epithelial tissue consistent with cyst material and keratin debris. One week postoperatively, the patient's eye remained quiet with the best spectacle-corrected visual acuity (BCVA) of 20/80 OD with no recurrence of the iris cyst (Figure 1(c)).

**Figure 2:**
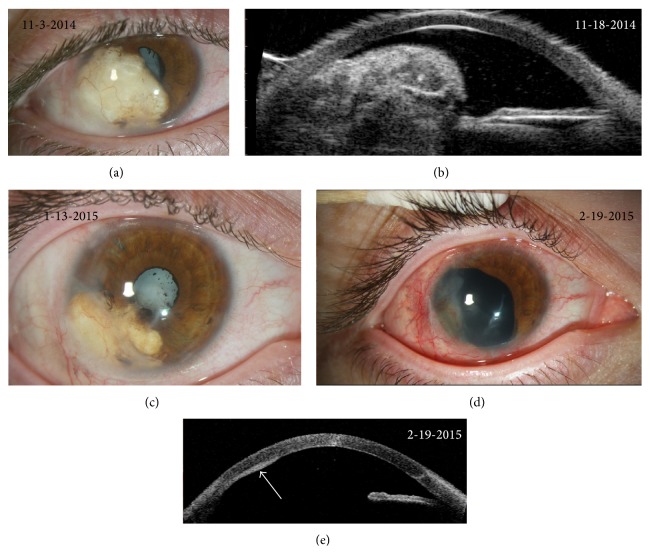
Clinical and diagnostic images from case 1: subsequent presentation. (a) Slit-lamp photograph illustrating recurrence of the iris cyst along the inferotemporal iris after the patient was lost to follow-up for 1.5 years. Posterior synechiae have developed and the pupil is largely obstructed by the iris cyst. A white cataract can be seen through the small pupillary opening. (b) Ultrasound biomicroscopic images demonstrating copious echogenic material within the iris cyst. Normal iris and angle structure are disrupted and the cyst abuts the ciliary body. (c) Slit-lamp photograph showing shrinkage of the iris cyst after repeat debulking of the cyst and intracystic injection of 5-FU. (d) Slit-lamp photograph four weeks after surgical excision of the cyst demonstrating a large sector iridectomy. The cataract was removed with a vitrector and the patient is aphakic. The temporal cornea demonstrates posterior opacity and vascularization in the area previously abutted by the cyst. (e) AS-OCT image four weeks after surgical excision of the cyst. The sector iridectomy is seen. There is increased reflectivity and thickening on the posterior cornea in the area previously adjacent to the cyst where neovascularization and scarring were identified by slit-lamp biomicroscopy (arrow).

The patient was lost to follow-up for 1.5 years until he returned in 2014 with increasing pain and light-perception vision in his right eye. The iris cyst had returned, emanating from the inferotemporal iris, and was white and opaque in appearance. The cyst once again abutted the corneal endothelium over a larger area than upon initial presentation and was associated with corneal neovascularization in this area. There was now 360 degrees of posterior synechiae and a white cataract had also developed in the interim (Figure 2(a)). Ultrasound biomicroscopic (UBM) images revealed a solid cyst filled with copious echogenic material that disrupted the normal iris and angle architecture and abutted the ciliary body (Figure 2(b)). He subsequently underwent repeat removal of contents of the iris cyst, lysis of posterior synechiae, and injection of 1000 mcg 5-FU in 0.2 cc of Viscoat into the cyst cavity. This procedure was done to reduce the size of the cyst and improve the patient's symptoms but was not anticipated to be definitive treatment based on recurrence to date. Pathological analysis of the cyst contents revealed acellular eosinophilic material suggestive of keratin debris.

**Figure 3:**
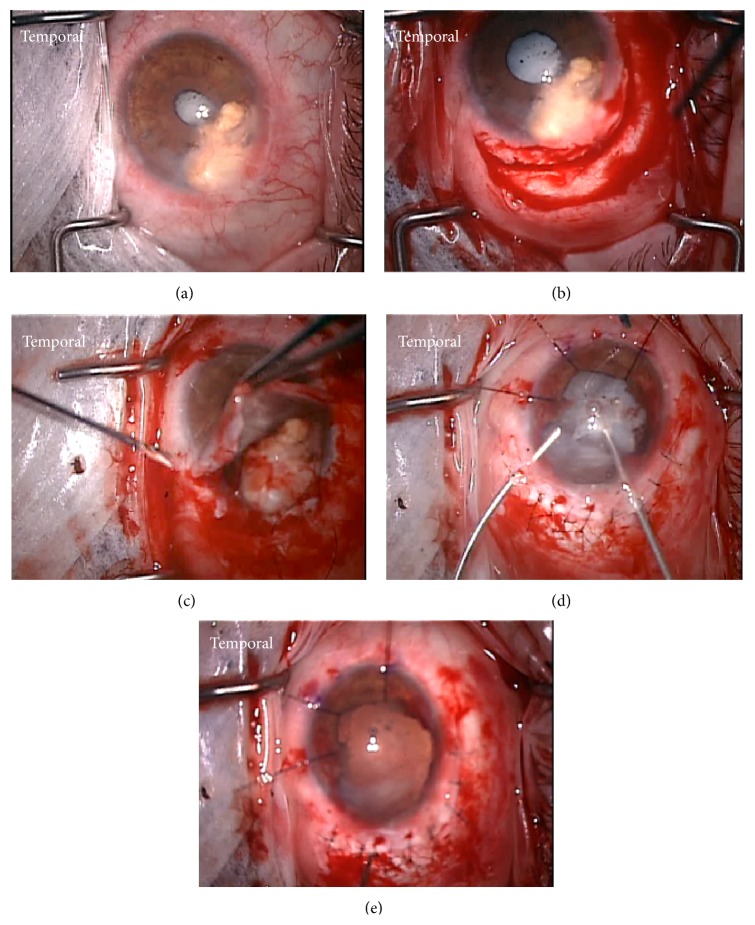
Steps of surgical procedure from case 1. (a) Intraoperative view of the iris implantation cyst along the inferotemporal iris. (b) A conjunctival peritomy and 180-degree scleral tunnel were fashioned along the temporal aspect of the cyst to facilitate access to and removal of the cyst. (c) The cyst was dissected from the posterior cornea using viscoelasticity. The cornea was then retracted to expose the cyst. Intraocular diathermy was applied along the iris adjacent to the cyst to reduce bleeding prior to performing a large sector iridocyclectomy. (d) The scleral incision was closed with nine 9–0 nylon sutures. After lysis of posterior synechiae, three iris hooks were used to retract the remaining iris tissue and the cataract was removed with an anterior vitrector. (e) Intraocular lens placement was deferred at the time of surgery until longer-term ocular stability could be demonstrated. The patient was left aphakic.

**Figure 4:**
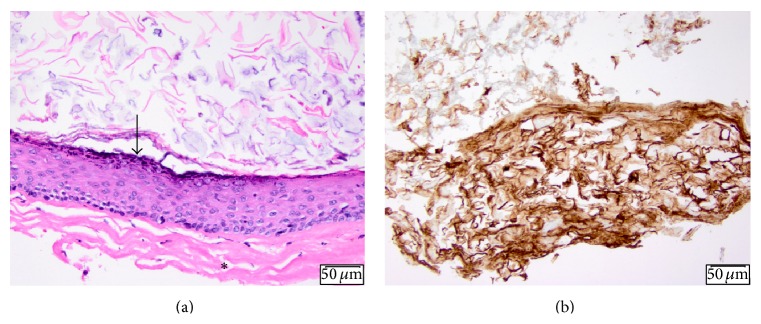
Histopathology images from case 1. (a) Iris implantation cyst lined by stratified squamous epithelium (arrow) with a large amount of keratin debris with the cyst cavity. Fibrosclerotic material is seen surrounding the cyst wall (asterisk). (b) Positive immunohistochemistry for pankeratin cocktail, confirming the cyst contents to be keratin debris.

The cyst reduced in size after this second procedure (Figure 2(c)), but the patient continued to experience severe pain and diminution of vision and opted to undergo complete resection of the iris cyst, lysis of synechiae, and cataract extraction three months later. An iridocyclectomy was performed to resect the entire cyst. The steps of the iridocyclectomy were as follows: After visual inspection of the cyst (Figure 3(a)), a conjunctival peritomy was performed along the temporal aspect of the cyst to expose bare sclera. A 180-degree scleral tunnel was fashioned from 5 to 11 o'clock (Figure 3(b)). The cystic lesion was dissected from the posterior cornea using viscoelasticity. The cornea was then retracted to expose the cyst (Figure 3(c)). Intraocular diathermy was applied along the iris at the edges of the cyst to reduce bleeding, and a sector iridocyclectomy and cyst removal were performed. The scleral incision was closed with nine 9–0 nylon sutures. Through peripheral incisions, the posterior synechiae were lysed. Three iris hooks were used to retract the remaining nasal part of the iris. Zonular instability occurred due to the cyst and its subsequent removal. The lens was removed with a vitrector as well as any vitreous that presented anteriorly (Figure 3(d)). Given the limited visualization and until longer term stabilization could be ensured, the patient was left aphakic (Figure 3(e)). The patient had postoperative hyphema and vitreous hemorrhage. However, three weeks later, his pain had decreased and spectacle-corrected visual acuity had improved to 20/400 with resolved hyphema and improving vitreous hemorrhage (Figure 2(d)). Postoperative AS-OCT showed the sector iridectomy and increased reflectivity and thickening on the posterior cornea in the area previously adjacent to the cyst, where neovascularization and scarring were identified by slit-lamp biomicroscopy (Figure 2(e)). Pathological analysis of the resected iris tissue revealed an epithelial lined cyst with keratin, keratohyalin granules in the epithelium, and subepithelial pigmentation (Figures 4(a) and 4(b)).

The patient will continue to be monitored for recurrence of the iris implantation cyst. While he remains early in his postoperative course, the use of a rigid gas permeable lens or insertion of a secondary intraocular lens with a penetrating keratoplasty can be considered to maximize his visual outcome.

## 3. Case 2

A 45-year-old male with a history of childhood penetrating ocular trauma from a scissor injury with subsequent repair in the right eye was diagnosed with an iris implantation cyst in 2002 at an outside institution. The cyst spontaneously collapsed and required no further treatment. After an episode of iritis and subsequent cataract development in the same eye, he underwent uncomplicated cataract surgery with posterior chamber intraocular lens implantation in 2007. In 2008, the cyst recurred and was aspirated and treated with laser therapy.

**Figure 5:**
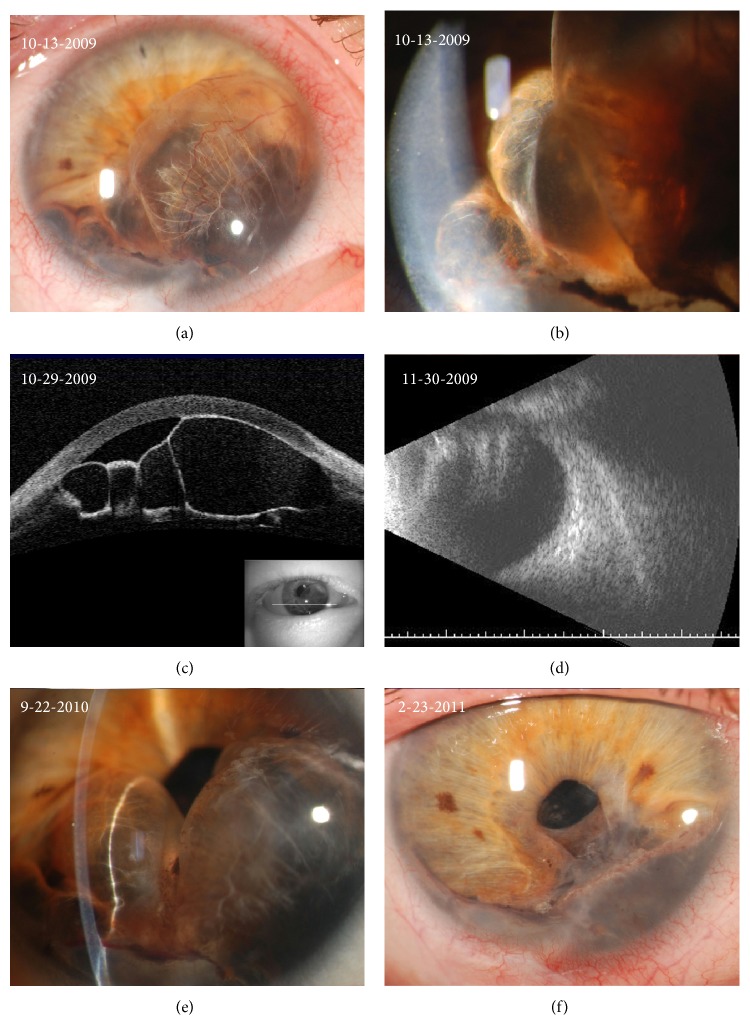
Clinical and diagnostic images from case 2. (a and b) Slit-lamp photographs illustrating multiple serous cysts emanating from the inferior iris, obscuring the pupillary opening, and apposing the corneal endothelium. Blood vessels and iris pigment can be seen along the cyst walls. The inferior cysts appear more opaque and homogenous as compared with the superior cysts. (c) AS-OCT showing 4 distinct cavities with low central reflectivity and highly reflective walls, confirming the fluid-filled nature of the iris cysts. The cysts can be seen abutting the corneal endothelium and normal iris architecture is disrupted. (d) B-scan ultrasound image confirming extension of the iris cysts into the vitreous cavity. (e) Slit-lamp photograph showing recurrence of multiple, serous iris cysts that once again appose the corneal endothelium and encroach upon the pupillary opening. (f) Slit-lamp photograph 1 year after cyst aspiration and second injection of 5-FU. There is complete regression of the cysts with residual iridocorneal adhesions.

His vision remained stable until he first presented to the Flaum Eye Institute in October 2009 with pain, decreased vision, and formation of several iris cysts. Visual acuity was 20/50 in the right eye, intraocular pressure was 19 mmHg, and anterior segment examination revealed multiple, vascular, fluid-filled cysts emanating from the inferior iris that were apposed to the corneal endothelium and obscuring the pupil (Figures 5(a) and 5(b)). Pigmented cells were seen floating within the cyst cavities. AS-OCT confirmed four multilobulated, fluid-filled cysts that disrupted the normal iris architecture (Figure 5(c)). B-scan indicated extension of the cysts into the vitreous cavity (Figure 5(d)). Four months later, the patient's vision further decreased to 20/400 and the cysts continued to rapidly enlarge, now occupying 50–60% of the anterior chamber. Given the cysts' significant growth and extension into the visual axis and posterior chamber, cyst contents were aspirated and 0.1 cc of 5-FU mixed with viscoelasticity at a concentration of 500 mcg/0.1 mL was injected into the cyst cavities. Two months postoperatively, vision improved to 20/25 with complete cyst regression.

However, three months later, the patient began to experience increasing pain and decreased vision and was found to have recurrence of multiple fluid-filled cysts (Figure 5(e)). He underwent repeat aspiration of cyst contents and intracystic injection of 5-FU in November 2010. Aspirated cyst fluid from both procedures contained macrophages and degenerated debris; fungal stains and cultures revealed no organisms and cytological studies were negative for neoplasms. One year postoperatively, his vision improved to 20/40 with no recurrence of the cysts to date (Figure 5(f)).

## 4. Review of the Literature and Discussion

### 4.1. Case Discussions

The first case describes the development and recurrence of a keratin-filled iris implantation cyst following penetrating ocular injury and posttraumatic implantation of intraocular cilia in the anterior chamber. Iris implantation cyst formation secondary to such an etiology is a rare occurrence [4, 6–11]. Histopathological analyses have shown these cysts to be of epidermoid as well as dermoid origin [10]. In our patient, prior penetrating trauma and iris prolapse permitted introduction of epithelial cells and cilia into the anterior chamber and caused damage to the iris tissue that was entrapped in the wound. Implantation of the base of cilia into the altered iris tissue likely allowed for survival and proliferation of the epithelium from the follicles and/or root sheaths of the implanted cilia along with the surface epithelial cells, triggering development of a keratin-filled, pearl cyst [4, 6–10]. The eye's response to retained cilia can be variable, ranging from acute inflammatory reactions to no complications, with the longest reported asymptomatic period being 32 years [6–10]. Our patient's eye remained quiet for two years after the initial injury before the onset of symptoms and cyst development. Although both embedded cilia were removed during the initial debulking of the cyst, recurrence was observed, suggesting that there were remaining epithelial cells embedded within the iris tissue that continued to actively proliferate despite treatment with an antiproliferative agent [6–10]. Conservative treatment measures were initially employed to achieve cyst regression; however, in light of the patient's unresolving pain, cyst recurrence, and impending risk of further complications, aggressive surgical intervention was ultimately required.

The second case demonstrates the development of recurrent, serous iris implantation cysts following penetrating ocular injury and cataract surgery. The initial traumatic injury likely permitted intraocular implantation of surface epithelial cells that remained dormant for nearly 35 years until cyst formation was first observed. Although the cyst initially spontaneously regressed, subsequent intraocular surgery may have either allowed additional epithelial cells to enter the eye and proliferate or reactivated the already implanted epithelial cells. Although aspiration and laser therapy were unsuccessful in preventing recurrence, addition of an antiproliferative agent allowed for complete cyst regression, and more extensive anterior segment surgery was avoided.

In both cases, the diagnosis of the iris implantation cysts was primarily clinical, but AS-OCT and ultrasound imaging were utilized to help further characterize the nature and extent of involvement of the cysts and guide the clinician's treatment approach. Final confirmation was obtained from histopathological analyses of resected tissue. We use these two cases to discuss the diagnostic and therapeutic challenges of posttraumatic iris implantation cysts and provide recommendations for the management of difficult cases.

### 4.2. Etiology and Presentation of Iris Cysts

Iris cysts are traditionally classified as primary or secondary cysts based upon their etiology [1–3]. Primary cysts can include posterior pigment epithelial cysts, iris stromal cysts, and free-floating/dislodged cysts. Secondary or acquired implantation cysts develop in the setting of ocular trauma, tumors, inflammatory conditions, parasitic ocular invasion, or prolonged use of topical miotics or prostaglandins [1, 2, 4]. Among secondary cysts, posttraumatic implantation cysts, as exhibited in the above cases, are encountered more commonly. These cysts arise from intraocular implantation of epithelial cells from the skin, conjunctiva, or cornea following perforating ocular trauma or intraocular surgery [1, 2, 5, 12–17]. Experimental studies have confirmed that introduction of corneal or conjunctival epithelium into the anterior chamber can trigger cyst formation [18–20]. Although the aqueous humor is thought to inhibit the survival of foreign cells within the eye, alteration of the aqueous by severe disease can allow these cells to survive [4, 21]. The vascular iris tissue can also create a suitable scaffold and environment for cell growth that permits successful implantation and proliferation of epithelial cells [1–5, 21]. Risk factors for posttraumatic epithelial implantation cyst development include prolonged postoperative hypotony, incarceration of the lens capsule or iris, and wound dehiscence or leak [1, 2, 4].

Secondary implantation cysts can present with symptoms such as pain, redness, decreased vision, and photophobia. Cyst development is typically preceded by inciting trauma, surgery, or inflammation and these lesions can arise many years, anywhere from 1 to 20 years, after the initial insult [1, 4, 21, 22]. Secondary implantation cysts have been described to take on three distinct appearances: serous cysts, pearl cysts, and epithelial downgrowth [1, 5, 20, 21, 23]. Serous cysts, as seen in case 2, are the most common among the manifestations and present as thin-walled, septate, fluid-filled, translucent structures, oftentimes with floating particles within the cyst cavity. They tend to develop large tumor diameters and induce iris atrophy and may invade the posterior chamber through iris erosion [2]. In contrast, pearl cysts, as seen in case 1, present as dense, opaque structures embedded within the iris stromal tissue that contain keratinous or inflammatory debris. They often develop in the presence of intraocular foreign bodies [1, 2, 21]. Epithelial downgrowth, a form of aggressive epithelialization of the anterior chamber, can appear not only as sheets but also as cysts or pearls and should remain on the differential when iris cysts are encountered [1, 2, 4, 5, 22, 23].

When assessing iris cysts, it is important to differentiate primary and secondary iris cysts from solid iris tumors. Tumors are typically solid, thick-walled lesions with irregular surfaces and borders while cysts are fluid or debris filled, thin-walled lesions with smooth surfaces and regular borders. Cysts tend to arise from the iris stroma or pigment epithelium while tumors displace the stroma or epithelium. In addition to these clinical characteristics, tumors are larger in size and more often associated with complications including vascularization, ectropion uveae, iris infiltration, pupillary distortion, cataract, and glaucoma development [1, 2].

Vascularity becomes important when distinguishing iris cysts from tumors. The presence of sentinel vessels and intrinsic vessels within the lesion is suggestive of solid tumors rather than cysts [2]. In our two cases, no sentinel vessels were observed and vessels were seen only on the walls of the cysts and not within. It is presumable that these vessels were a result of the proliferation of existing iris vasculature rather than abnormal vessel growth. The vascular pattern of lesions can also be assessed in comparison to the normal vascular pattern of the iris to distinguish between cystic and malignant lesions as tumors exhibit disorganized vasculature. Iris fluorescein angiography is a diagnostic tool that can be used to identify vessel leakage or disorganized vascular networks, both of which are more suggestive of malignancies; however, angiography is not commonly employed due to its invasive nature [2]. Intracameral antivascular endothelial growth factor therapeutic agents, particularly bevacizumab, have emerged as treatment modalities for anterior segment vascularization given their antiproliferative and antiangiogenic properties. Experimental and clinical studies have demonstrated vessel regression with bevacizumab in cases of corneal and iris neovascularization [24]. These agents may therefore play a role in facilitating abnormal vessel regression that arise with iris tumors; however in our two cases, no abnormal vascular growth was observed in the implantation cysts that warranted antiangiogenic therapy.

### 4.3. Diagnostic Modalities

The diagnosis of primary or secondary iris cysts is primarily clinical but concurrent use of imaging technology can facilitate diagnosis and therapy. UBM has remained the imaging modality of choice for iris cysts as it has been shown to provide high-resolution images that permit an accurate assessment of the dimensions, shape, internal features, and location of these lesions. AS-OCT is a noninvasive imaging tool that can also be used to visualize anterior segment lesions. By utilizing sound waves, UBM is able to better penetrate through various ocular tissues, while AS-OCT, due to the use of a light source, is limited by its inability to penetrate through the opaque sclera or iris pigment epithelium, which obscures more posterior structures [25–28]. UBM images clearly depict the relation of these lesions to surrounding structures, such as the overlying cornea, underlying lens, or ciliary body, as well as extent of involvement, which can help clinicians determine if conservative or more radical surgical approaches are necessary [1–3, 13, 25–28]. Studies comparing the utility of AS-OCT and UBM for the evaluation of anterior segment lesions, specifically tumors, have revealed that UBM is superior to AS-OCT in visualizing larger, highly pigmented lesions and assessing posterior or ciliary body extension. AS-OCT can, however, better evaluate smaller lesions on the anterior iris or superficial, nonpigmented lesions, such as conjunctival tumors. Both can accurately differentiate solid and cystic structures [25–28]. B-scan ultrasonography can also be utilized to visualize the extent of anterior and posterior segment involvement of iris cysts. Immersion B-scan or UBM provide higher resolution images and have been shown to be superior for assessing ciliary body involvement [25].

On UBM, secondary implantation cysts have distinctive findings depending on their subtype. Pearl cysts are described to have three layers with differing reflectivity: a moderately reflective epithelial cyst wall, an intermediate layer with lower reflectivity composed of degenerated epithelial cells, mucus, and inflammatory debris, and a central, highly reflective core filled with keratinous debris. Serous cysts are also described to have moderately reflective cyst walls but instead have primarily anechoic central cavities [4, 13, 26].

In our cases, AS-OCT provided helpful visualization of corneal findings as well as cyst contours and relation to surrounding structures. In case 1, the healed corneal scar from the patient's traumatic injury was visible and high reflectivity from the surface of the lesion along with internal reflectivity confirmed the solid nature of the lesion (Figure 1(b)). Postoperatively, thickening as well as high-reflectivity of the posterior cornea along the site of the excised cyst was clinically correlated to corneal scarring and neovascularization in that area (Figure 2(e)). AS-OCT images from case 2 revealed four distinct cavities with empty lumens and highly reflective walls, confirming the serous nature of these lesions (Figure 5(c)). In both cases, the proximity of the cysts to the posterior cornea and the obscuration of normal iris and angle architecture were easily appreciated with the AS-OCT.

UBM imaging in case 1 was used to further characterize internal features of the cyst. Images demonstrated copious echogenic material in the central cavity (Figure 2(b)), which was not clearly delineated on AS-OCT. UBM images also illustrated extension of the cyst to the border of the ciliary body. Preoperative identification of the cyst's extension and internal features aided the clinician in planning the operative procedure, including the location of the incision site, extent and degree of the iridocyclectomy, and cataract extraction. In case 2, a UBM was not available and therefore B-scan imaging was used to confirm extension of the cysts into the vitreous cavity.

Final diagnosis of implantation cysts is usually confirmed with histopathological analysis of resected tissue [1, 2, 12]. Pearl cysts have concentric layers of stratified squamous epithelium while serous cysts are thin walled structures lined with flat, atrophic epithelium. Pearl cysts contain inflammatory or cellular exudates, mucin, or keratin debris while serous cysts are fluid-filled, sometimes with interspersed particulate matter [1–3, 12].

In case 1, histopathological analyses of the cyst contents after the initial debulking procedures revealed only keratin debris and no epithelial tissue but after complete resection of the cyst, stratified squamous epithelium and keratin debris were seen (Figures 4(a) and 4(b)), confirming a keratin-filled, pearl cyst. In case 2, cyst fluid was aspirated but the cyst walls were not excised; therefore no epithelial cells were observed but analysis of aspirated fluid revealed macrophages and degenerated debris, confirming serous cysts. In both cases, the initial procedures (debulking in case 1 and aspiration/laser in case 2) did not remove the epithelial cyst walls in their entirety thus providing an opportunity for eventual recurrence of the cysts.

### 4.4. Treatment Options

Implantation cysts, unlike primary cysts, can be progressive and destructive in nature. Smaller cysts can be merely observed over periods of time, but larger cysts often require expedited and aggressive treatment, especially when they cause complications such as persistent pain, obstruction of vision, iris bombe, pupillary obstruction, cataract formation, corneal decompensation, glaucoma, or uveitis. Unfortunately, despite treatment, long-term visual prognosis remains poor in many cases, largely in part due to cyst recurrence and secondary complications [1, 3, 29–31].

Surgical management of penetrating trauma is important in avoiding formation of implantation cysts and their secondary complications. Careful and prompt exploration of the extent of injury should be performed along with rapid closure of the wound with minimal collateral damage and disruption of normal ocular anatomy. Often prolapsed tissue can develop epithelial coverings. This tissue can be treated with a cryoprobe and the epithelium can be subsequently gently debrided prior to wound reapposition to avoid intraocular seeding of foreign epithelial cells. Wound leak is an important postoperative complication of open globe injury repair and persistent wound leak could contribute to entry of epithelial cells into the eye. Meticulous and prompt wound management must therefore be employed to reduce this risk [32].

When the decision is made to treat, a spectrum of approaches can be considered, ranging from conservative management to aggressive surgical excision. Cyst puncture and debulking coupled with aspiration of cyst contents can be attempted with careful attention as to not allow expulsion of contents into the anterior chamber. Viscoelastic dissection of the cyst, cauterization, diathermy, electrolysis, and cryotherapy can also be implemented [1, 29, 30, 33–35]. Intracystic injections of sclerosing agents or cytotoxic agents such as phenol, saline, iodine, 50% dextrose, ethanol, or mitomycin-C (MMC) can be used to halt cyst growth [1, 36–39]. Argon laser photocoagulation and Nd:YAG laser iridocystotomy can be employed with the above methods to ablate and devitalize cyst wall epithelial cells, leading to cyst shrinkage [3, 14, 21, 23, 31, 40–45]. Many of these conservative options are often used in combination to achieve maximal long-term success; however, recurrences can still occur [1, 15, 29, 34, 43, 46].

In the setting of recurrence or for high-risk cyst-induced complications, surgical excision can be performed and the preferred surgical approach is determined based on the extent of involvement of the cyst. Options can range from en bloc resection, sector iridectomy, to extensive inner lamellar corneoscleral iridocyclectomy requiring corneoscleral grafts [1, 2, 4, 5, 11, 23, 29, 43, 47–50]. In case 2, multiple conservative measures including cyst aspiration, laser therapy, and injections of antiproliferative agents ultimately led to complete cyst regression without the need for more extensive surgery. However, in case 1, an iridocyclectomy was performed due to cyst recurrence despite conservative debulking procedures as well as the cyst's large size and anterior chamber angle involvement.

Both conservative and surgical interventions do carry the high risk of cyst rupture, typically due to direct manipulation of cyst tissue. Cyst rupture can cause further intraocular seeding and spread of epithelial cells and formation of intractable, diffuse epithelial downgrowth, which carries a grave prognosis [22, 46]. Often, en bloc excision can be an advantageous treatment option for cysts. Although this procedure is destructive and can cause collateral damage to adjacent ocular structures, it does allow for the most complete treatment and avoids excessive manipulation of cyst tissue, which can reduce the chances of inducing diffuse epithelial downgrowth [29]. Complications of iridocyclectomy specifically include hyphema, iridodialysis, vitreous loss, lens subluxation, retinal detachment, infection, wound dehiscence, wound leak with hypotony, and cataract formation [49–51]. While surgery often allows for the most complete eradication of such lesions, it is also associated with higher rates of complications and longer recovery times. Minimally invasive and more conservative approaches are therefore typically recommended as first-line treatment before invasive anterior segment surgery is attempted. However, all treatment options must be employed carefully to minimize potential vision loss.

5-FU is a pyrimidine-analog antimetabolite commonly used in glaucoma filtering surgeries and bleb revisions, dacryocystorhinostomy procedures, pterygium surgeries, conjunctival neoplasias, and vitreoretinal surgery, due to its antiproliferative and antiscarring properties [52, 53]. Recently, there have been several reports exhibiting the use of 5-FU for successful treatment of diffuse epithelial downgrowth [54–57] but use of this medication in the management of iris implantation cysts has not been previously reported. Iris implantation cyst occurrence has been attributed to the prolific nature of the epithelial cells along the wall of the cyst cavity [3]. In both cases, we therefore injected 5-FU in ophthalmic viscoelasticity at two separate times to destroy any proliferating epithelial cells within the cyst cavities and surrounding iris tissue. In case 1, while we did not achieve permanent resolution with an antiproliferative agent, the injections did allow for intermittent reductions in cyst size in conjunction with cyst debulking and aspiration. We hypothesize that the size and density of the keratin-filled cyst likely precluded complete penetration of the drug and limited its efficacy. Ultimately, surgical intervention was necessary as our patient was suffering from a blind, painful eye and was at risk for further complications given the rapid enlargement of the cyst. However, as seen in case 2, injections of 5-FU did allow for complete cyst regression, and we hypothesize that the thinner walls and fluid-filled nature of these implantation cysts permitted better penetration of the antimetabolite into the cyst cavity and iris tissue.

Intracystic injection of MMC, another antiproliferative agent, was first used by Kawaguchi et al. to achieve iris cyst regression [36]. MMC is a dual DNA cross-linking as well as RNA and protein synthesis inhibitor that has been used as an ocular antiproliferative agent in glaucoma filtration surgery, pterygium surgery to inhibit fibroblast proliferation, refractive surgery to reduce postoperative haze, primary acquired melanosis, conjunctival melanoma, ocular cicatricial pemphigoid, and corneal and conjunctival dysplasias. MMC is thought to cause damage to epithelial and goblet cells that line cystic cavities and facilitate cyst regression through this mechanism [58]. Use of MMC has been associated with adverse effects such as conjunctival irritation, tearing, and superficial punctate keratopathy and animal studies have shown additional effects including wound leak, endothelial damage, ocular hypotony, neuroretinitis, and endophthalmitis [36, 58]. As such, careful attention must be taken to not allow MMC to leak into the anterior or posterior chambers during administration. 5-FU is overall well tolerated in the eye with no reports of corneal and retinal toxicities and only minor conjunctival tissue reactions in animal studies [53].

MMC is a viable, conservative treatment option for iris implantation cysts. However, given our prior experiences with 5-FU, high safety profile of the agent, and previous reports in the literature of its success in treating epithelial downgrowth, we felt its use was appropriate for our patients. In addition, use of this medication in the management of iris implantation cysts has not been previously reported and with the favorable results seen in our two cases, we believe it can be considered for the treatment of iris implantation cysts prior to more aggressive surgical intervention.

## 5. Summary

In summary, our cases illustrate the development and recurrence of posttraumatic iris implantation cysts with uncommon etiologies and underscore the challenges in the accurate diagnosis and management of this condition. From our experiences and a review of the literature, we recommend the use of AS-OCT and UBM along with histopathological analyses of resected tissue to facilitate accurate diagnosis. Intracystic injection of 5-FU is a novel treatment that can serve as an effective adjunctive option prior to aggressive surgery to reduce treatment complications and improve visual outcomes. Careful surgical planning is needed in such cases to ensure complete cyst resection and minimize complications.
